# Supporting clinician educators to achieve “work-work balance”

**Published:** 2016-10-18

**Authors:** Jerry Maniate, Deepak Dath, Lara Cooke, Karen Leslie, Linda Snell, Jamiu Busari

**Affiliations:** 1 Wilson Centre & Department of Medicine, Faculty of Medicine, University of Toronto, ON; 2 Department of Surgery, McMaster University, QC; 3 Department of Clinical Neurosciences, Cumming School of Medicine, Calgary, AB; 4 Centre for Faculty Development, University of Toronto, ON; 5 Centre for Medical Education, McGill University, QC; 6 Faculty of health, medicine and life sciences, Maastricht University, Netherlands

## Abstract

Clinician Educators (CE) have numerous responsibilities in different professional domains, including clinical, education, research, and administration. Many CEs face tensions trying to manage these often competing professional responsibilities and achieve “work-work balance.” Rich discussions of techniques for work-work balance amongst CEs at a medical education conference inspired the authors to gather, analyze, and summarize these techniques to share with others. In this paper we present the CE’s “Four Ps”; these are practice points that support both the aspiring and established CE to help improve their performance and productivity as CEs, and allow them to approach work-work balance.

## Background

The professional life of a Clinician Educator (CE) is a challenging one, filled with competing responsibilities. Like other academic physicians, CEs balance their career trajectory (in this case, the educational stream) with the clinical, scientific, and administrative duties demanded of a physician in academic practice. A CE is “a clinician active in health professional practice who applies theory to education practice, engages in education scholarship, and serves as a consultant to other health professionals on education questions and issues.”^1^ Therefore, in addition to clinical practice, CEs teach, conduct research and scholarship, develop educational innovations (through curriculum development and implementation), perform education consultations, and carry out administrative roles, such as program coordination.^2^ With these competing professional responsibilities and demands, many CEs face challenges trying to achieve “work-work balance.”^3^

### Striving for “work-work balance”

CEs have a three-way work balance to achieve: they must balance their clinical work with their education activities that then consist of scholarly work (education activities including teaching, curriculum development, and innovation); education scholarship; and leadership. Steady scholarship is the metric that universities generally use to mark CEs for promotion. However, the time available to develop publishable work is often dependent on clinical and other education responsibilities that wax and wane, sometimes unpredictably and uncontrollably. CEs and their universities have to acknowledge that meaningful scholarship takes time. They must jointly advocate for the investment of time required for education scholarship, especially in the face of last-minute requests to develop workshops, teach, mentor, design curricula, provide consultation to others, or take part in clinical activities. These education activities are crucial aspects of the CE role. In fact, it is arguably these same activities that hone a CE’s skills; therefore scholarly activities must be managed in conjunction with clinical work and scholarship.

In 2012, Snell used the phrase “work-work balance” to “describe the challenge CEs have in finding equilibrium between their education and scholarly endeavors and the obligations of clinical practice.”^3^ Recently, “work-life integration”^4^ has been used to describe the interdigitation of personal and professional roles and activities. Indeed, the advent of technological and cultural changes has made it easier for CEs to work from home, to remain in communication with work while attending to family matters, and to be more flexible about when and where they do their educational work. CEs must take into account these unique personal and relational factors that determine how they allocate time to their activities. The degree of integration or separation between work and family matters is a dynamic and personal process that requires good judgment; therefore, work-life integration does not lend itself to easy generalizations and frameworks. “My family comes first” is a good first approximation, but more principles and techniques may be found when others take up research in this field. In the meantime, this paper focusses on the more generalizable *concepts, skills, and competencies* that allow CEs to navigate the various work responsibilities that they take on in their professional lives.

### Sharing CE Experiences

At the 2013 Royal College of Physicians and Surgeons of Canada (RCPSC) International Conference on Residency Education (ICRE), an interactive session facilitated by a panel of international speakers allowed CEs to share their experiences on how they approach work-work balance. The participants offered suggestions and generated discussions to help CEs be more effective in all their roles, including scholarship. It was clear that although CEs come from a variety of clinical and professional backgrounds, as well as different higher education institutions, they described many common experiences and techniques for dealing with work-work balance. Participants decided at the end of the session that they would take the opportunity to transform their discussion into scholarship. Several (the authors) developed the content in this paper from what they learned at the panel discussions, their own experiences, and a review of the literature. The paper is organized around a simple mnemonic framework developed by one author (JM). The Hamilton Integrated Research Ethics Board waived a full review of this work when methodology was described and the manuscript presented.

So how might CEs achieve better work-work balance? This paper presents the CE’s “Four Ps” - practice points that are intended to support the aspiring and established CE in order to help enhance their performance and productivity as CEs and allow them to approach “work-work balance” in the context of their lives.

### First P - Prioritize activities

Align! (Jason Frank, Clinician Educator)

To be able to prioritize their activities, CEs should develop their vision, track and revise their activities, consider how opportunities for new work align with their vision, and aim to develop a portfolio of successful projects. CEs should use deep reflection to craft their personal visions that will determine how their professional activities are prioritized. Because vision is so important, CEs need a personal vision document. Early on, they must be able to articulate clearly their visions as they seek advice from diverse mentors, such as productive researchers, accomplished leaders, senior medical educators, and respected clinicians. Good mentors will alert CEs of available work opportunities or provide opportunities to allow CEs to grow.

What are you letting go of, in order to add this new “something” to your workload? (Karen Leslie, Clinician Educator)

Vision documents will require periodic review, and CEs should review and revise their career trajectories regularly to ensure alignment with their vision. This will allow them to recognize and take advantage of work opportunities that fit their vision. CEs can discipline themselves to log their educational contributions in their CVs and teaching dossiers in a timely fashion so that these documents are accurate reflections of their work. They can then schedule time to review their current activities and assess them for relevance, priority, and alignment with their responsibilities as new opportunities arise. This reflective process will allow CEs to refocus on high-yield and high-quality activities that will strategically align their personal vision and their career trajectories. They will also be able to take on work that will benefit their patients, their learners, their peers, and their institutions, without subverting their vision.

Saying no is easy. Just open mouth, say no, close mouth. (Meredith Marks, Clinician Educator)

Most of a CE’s current medical education projects will likely have been chosen by design. That is, they will either have been chosen to align with long-term goals, or they are meaningful projects - assigned or stumbled into - that have modified future goals and are worth continuing. However, CEs with multifaceted work-lives may feel pressure from their multiple collaborative partners, or may themselves feel obliged, to say “yes” to new projects and activities, even when they may not fit the desired work trajectory. A CE must be prepared to say “no” to new opportunities if they are not aligned with existing roles and responsibilities or future goals and plans.

Declining some projects and opportunities will allow CEs to maintain their current work and to develop themselves and their skill-sets in familiar settings until they feel ready to transition to other phases of their work lives. It is also more important to a CE’s career and self-satisfaction to complete projects well rather than to become involved with myriad projects. Maintaining a focus by prioritizing one’s activities is important to being recognized as someone who takes on and finishes projects well. This capacity to “deliver” paints a CE’s portfolio and encourages others to offer CEs opportunities that are more appropriate.

### Second P - Plan ahead

I’ll be at my Carlisle campus if you need me. (Deepak Dath, Clinician Educator)

CEs can successfully organize their professional lives by scheduling blocks of time for their educational work, by using technology wisely when communicating, and by realizing the multiple benefits that many of their activities afford.

CEs often pursue many different activities - clinical, educational, and personal - and thus, they need to pay close attention to scheduling in order to be productive with their time. Scheduling is described as “the art of planning your activities so that you can achieve your goals and priorities in the time you have available.”^5^ Thus, for many CEs, scheduling is comprised of: 1) decisions that must be made on how and where to spend their limited resources of energy and time; while 2) balancing tensions from numerous directions in their professional lives (including clinical pressures, research, teaching and administrative tasks); and 3) simultaneously trying to protect the personal aspects of their lives, which may include their family and personal interests. CEs will be better overall organizers, and will balance their myriad responsibilities better when they develop their scheduling skills.

Successful schedulers have insight into their work habits and available resources. First, CEs should schedule non-negotiable blocks of time for scholarly work, off-site if possible. Doing so will allow them uninterrupted time for necessary reading, research, educational development, and academic writing. If there is no opportunity to work offsite, CEs can close their office doors for their scheduled blocks to avoid distractions - remember, “out of sight means out of mind.” Working from a different (non-clinical) office may offer the synergy of working with education colleagues. Working from a home office - like the “Carlisle campus” - has the advantage of being around family or being able to transition immediately to family activity without having to commute home. Some savvy educators schedule work into naturally uninterruptible blocks of time like during airplane flights, an evening at a conference, or very early in the morning; others find these times impossible for conducting work.

When requests do come to participate in new activities, ensure that they do not erode “protected” time. Saying “let me check my calendar and get back to you,” allows CEs to check the new activity’s alignment with their goals, to ask others for their advice, and to either clear schedule space or decline gracefully.

We are drawn to the gentle buzz and flashes emitting from our mobile devices. Instead of making us more productive and freeing us, we seem to be distracted and chained to them. (Jerry Maniate, Clinician Educator)

Technology now offers too many ways to remain connected. CEs must develop their communication patterns by considering how different modes of communication will help them and their colleagues. They should also ensure that others know how and when to best communicate with them. Commonly, more involved communication that requires transmission of significant content still uses email; however, it is also one of the most destructive distractors, especially when a CE is on “protected” time. It is often not enough to try to ignore email. Consider maintaining focus by creating email-free periods, turning off email notification on smartphones and closing email programs during protected time for reading and writing.

Much good work is lost for the lack of a little more. (Edward H. Harriman, American Businessman)

Activities yield greater benefit when they are mined by pairing (double dipping) or tripling (triple dipping) the outcomes, saving both time and effort. For instance, when taking on a new medical education project, strategically plan how to fold in a scholarly component that could merit dissemination in publication. If implementing a new assessment method for competency based medical education (medical education work; first dip), pair it with a validation study for the assessment instrument (research; second dip) and invite and supervise medical students and residents in the research component (teaching and mentoring; third dip). This approach to alignment of activities can inform (but should not necessarily constrain) decisions to take on new opportunities as they present themselves.

### Third P - Persist with your passions

First, identify what makes you tick. Feel it and embrace it before you set out to get it. Then, stay focused as if nothing else matters; don’t let go of the feeling until you’ve achieved the goal. That is passion. (Jamiu Busari, Clinician Educator)

Passion is the very personal quality that drives a CE, requires some effort to maintain and is necessary to prevent a CE’s work from sputtering, smouldering, or failing.

Passion is clearly not a measurable quantity, but it is a basic, emotional driving force for effort and activity. Passion for work can be defined as “a strong inclination toward an activity that individuals like (or even love), that they find important, in which they invest time and energy and which comes to be internalized in one’s identity.”^6^ CEs should use reflection and insight to identify and describe what it is they are passionate about. Acknowledgement of this passion requires courage, but builds confidence and encourages action. Seeing a project or activity through to completion requires the perseverance that comes from a passionate drive. A CE’s passion may be more difficult to see when it is directed towards an overall good, instead of towards the details of the task-at-hand. For instance, some CEs might suffer through the anxiety of public speaking since they are passionate about creating and delivering faculty development workshops that require high-level public speaking.

CEs slog through work that they feel is either assigned or necessary because of position, instead of work that they have specifically chosen to do. Even when applying themselves to their chosen work, CEs must get through “the Dip” – that low point where the initial euphoria of novelty has worn off and the benefits of a large project have yet to materialize.^7^ During these times, CEs can challenge themselves to define how the work that they feel is a daily drudgery is actually in the service of their passions. A positive outlook on work is correlated with better outcomes.^8^ Successful work then has a positive and reinforcing effect on how people view their work, which may enhance intrinsic motivation. Motivated workers find it easier to apply themselves to their work and find more meaning in the work that they do, maintaining their passion.

Limited time and competing interests will conspire to reduce a CE’s productivity on all but those projects about which they are most passionate. Projects without passion are easily ignored to lie dormant, expire, cause loss of resources, or disengage collaborators. Persistence and passion are necessary to drive the project forward during the difficult times. CEs must find ways to reinforce those factors of the project that generated passion so that they can persevere through long or complex projects.

### Fourth P - Partner with others

Teamwork is the capacity to work together with others towards a common objective. It involves the ability to harness and direct individual accomplishments towards the common good of all. It is said to be the catalyst that drives common people to attain uncommon results. (Jamiu Busari, Clinician Educator)

CEs often work in teams, leading their assistants, identifying potential colleagues, obtaining and providing mentorship, and planning succession.

Many CEs have assistants with whom they can work towards achieving the CE’s professional vision. CEs should ensure that they share credit for successes with their assistants and take responsibility for failures since they are ultimately accountable for the execution of their work strategies. Setting joint expectations, timelines, protocols, and policies for tasks and communication will smooth operations and foster reciprocal trust. Skilled, protective assistants can provide a necessary buffer to minimize interruptions to a CE’s workflow.

*Sharon, hold my calls, please. I’m on Med Ed time.* (Deepak Dath, Clinician Educator)

Assistants can be natural guardians of a CEs time - delaying, reducing or completely blocking interruptions. CEs can also ask their assistants to take over some administrative duties, documentation, simple scheduling, and preparing meeting details. Finally, there are some tasks that assistants can get others to do for CEs, such as organizing a literature search. Supervision and collaboration with assistants should be a dynamic process from which both CEs and their assistants continue to learn.

CEs who lead projects should aim to identify collaborators who can share parts of a project and complement the existing expertise of the proposed team with a diversity of ideas and competencies. Make the most of these collaborators by giving them some latitude to achieve the task the way they feel is best, according to their expertise. However, with leadership comes the responsibility to rein in individuals who take a project in different directions, motivate some individuals, hold everyone (including themselves) accountable, and occasionally say “no.”

*Given how complex our personal and professional lives are, having different mentors participate as advisors for focal areas can provide us with the needed broader perspective on decision-making during our life journey.* (Jerry Maniate, Clinician Educator)

Effective mentorship plays a significant role in a CE’s success^9^ - providing inspiration, advice and feedback, and opportunities for growth. CEs should strategically seek out several mentors with whom to cultivate relationships. These mentors can serve as a “personal board of directors,”^10^ guiding CEs in the different areas of their professional work and providing an important developmental network. Mentors can provide CEs with broad oversight of their career trajectories and review their current work with an eye for assisting them with the tough work of pruning and refocusing. Proactively schedule meetings with mentors to review current activities, overall productivity, and to identify future opportunities for growth and development.

Multipliers are genius makers. Everyone around them gets smarter and more capable. People may not become geniuses in a traditional sense, but Multipliers invoke each person’s unique intelligence and create an atmosphere of genius – innovation, productive effort, and collective intelligence.^11^

CEs can benefit greatly from having mentors, and in turn should identify their colleagues who look to them for guidance. Even junior CEs can offer their mentees opportunities and projects that are not necessarily in alignment with their own current career trajectories. As CEs take on new roles, they can strategically pass previous roles to junior colleagues who can use these roles to build new competencies, to position themselves for roles that align with their goals, and to add scholarly contributions to their CV.

CEs who work collaboratively to build capacity in others will start to recognize candidates for their succession. Succession planning is a process for identifying and preparing individuals to assume leadership roles as these positions become available in a department or organization.^12^ CEs can identify potential successors early from among the junior faculty members for whom they provide mentoring, introductions, and opportunities.

## Conclusions

The professional life of a CE is challenging. The pressures of academic, educational, and clinical demands will always threaten balance. This paper shares the concepts, techniques, and competencies that successful CEs have found to help maintain the balance in their professional work. This attention to “how we practice” is lacking in medical education and could be addressed in postgraduate training or in a mentorship process for new CEs. CEs constitute a small group of educators who have much to learn from each other. They are organizing on national and international levels at conferences and online. ICENet^2^ is an example of an organization that organizes CE conferences in collaboration with major medical education conferences and provides an online, interactive blog. Aspiring and successful CEs are encouraged to network and learn from each other. The “Four Ps” – Prioritize, Plan, Persist, and Partner - offer a mnemonic framework that CEs can use to think about how they maintain their functionality, productivity, and passion while they balance the facets of their professional lives.

## Figures and Tables

**Table 1:** A repertoire of several ways to say “no”

Saying No
A 3-step approach: open mouth, say no, close mouth
We are close enough that when I say no I know you won’t take it personally.
If I were to say no to your request, who else do you think could help you?
My plate is so full at the moment that it wouldn’t be fair to you or to me if I took this on.
I trust you will understand when I say no.
I am flattered you would come to me for help: unfortunately, my schedule can’t handle it, right now.
I cannot free up any time (right now, today, this week…) – if it’s urgent you should probably find someone else this time.
I have only (5, 10, 15) minutes to spare now – can I show you a few things to help you take care of it yourself?
I would like to help but I must stay focused on my goals.
Can it wait until (time / date)?
If it will only take 5 minutes, I can do it now.
I am juggling a lot of tasks now: what do you think I should remove from my “to-do” list to help you?
I am sorry but I have another commitment.
Please see my assistant to organize that appointment.
Please clarify what exactly it is you are asking.
We have (time – e.g. 15 minutes) so what is/are the most important issues you want to address today?
This will work best for me in a face-to-face conversation – shall we book one?
